# Gait and lower limb muscle strength in women after triple innominate osteotomy

**DOI:** 10.1186/s12891-015-0524-3

**Published:** 2015-03-24

**Authors:** Sjoerd Kolk, René Fluit, Jim Luijten, Petra JC Heesterbeek, Alexander CH Geurts, Nico Verdonschot, Vivian Weerdesteyn

**Affiliations:** Department of Rehabilitation, Donders Institute for Neuroscience, Radboud University Medical Center, P.O. Box 9101, 6500 HB Nijmegen, The Netherlands; Laboratory for Biomechanical Engineering, University of Twente, P.O. Box 217, 7500 AE Enschede, The Netherlands; Sint Maartenskliniek Research, Hengstdal 3, 6522 JV Nijmegen, The Netherlands; Orthopaedic Research Laboratory, Radboud Institute for Health Sciences, Radboud university medical center, P.O. Box 9101, 6500 HB Nijmegen, The Netherlands

**Keywords:** Triple pelvic osteotomy, Hip joint, Walking, Congenital hip dislocation, Biomechanics

## Abstract

**Background:**

In adult patients with developmental hip dysplasia, a surgical procedure (triple innominate osteotomy) of the pelvic bone can be performed to rotate the acetabulum in the frontal plane, establishing better acetabular coverage. Although common clinical hip scores demonstrate significant improvements after surgery, they provide only overall information about function. The purpose of this study was to quantify the long-term outcome of triple innominate osteotomy in more detail using gait analyses and muscle strength measurements.

**Methods:**

We performed gait analyses at self-selected walking speed as well as isometric hip and knee muscle strength tests in twelve women who had undergone a unilateral triple innominate osteotomy (age: 34 ± 12 y, time post surgery: 80 ± 18 m). We compared the results to reference values obtained from eight healthy peers (age: 33 ± 10 y).

**Results:**

The patients exhibited slight asymmetries in step length (smaller steps) and stance time (longer stance) as well as lower hip abduction moments in the operated limb in early stance compared to the non-operated limb. However, there were no differences in gait compared to healthy controls, even though the patients showed reduced bilateral hip abduction strength compared to controls.

**Conclusions:**

Our results indicate that the patients’ gait pattern had generally recovered very well, despite slight asymmetries in spatiotemporal parameters. Subtle deviations in hip abduction moments were observed during gait, whereas hip abduction strength was substantially reduced. Hence, the patients walked at a higher percentage of their maximal capacity. They may, therefore, be prone to fatigue and adopt compensatory gait strategies more quickly than healthy peers when walking long distances.

## Background

Developmental dysplasia of the hip is characterized by a shallow, obliquely-oriented acetabulum and malposition of the proximal femur. Together, these anatomical deformations lead to an increased local load because of a decreased weight-bearing area [1]. This is painful for patients, can cause secondary osteoarthritis, and limits patients in their daily activities. To relieve the symptoms, a surgical procedure (Triple Innominate Osteotomy; TIO) can be performed [2,3]. In this surgery, the acetabulum is rotated in the frontal plane to increase its coverage over the femoral head [4]. This procedure reduces pain and further joint degeneration and, thereby, greatly improves the quality of life of patients [5]. In addition to pain relief and radiographic improvement, functional outcome is also important in determining the success of hip dysplasia surgery.

Most patients who underwent TIO achieve good to excellent functional results based on clinical functional scores [4,6,7]. However, drawbacks of these scoring systems are that they are subjective [8], may have ceiling effects [9], and only provide information about the overall level of function. They do not allow one to assess which joints or muscle groups underlie functional abnormalities. Thus, in order to obtain a quantitative and objective evaluation of function after TIO, it is necessary to employ more detailed and quantitative measurement techniques such as gait analysis [10-13]. In addition to gait analysis, muscle strength measurements are valuable to assess the biomechanical properties of the operated limb [14].

To our knowledge, there have been no reports on the impact of TIO at such a detailed functional level. The information obtained from gait analysis could provide a better assessment of the long-term effects of this surgery. It can be used to inform the, usually young-adult, patients about their expected walking ability after the operation [15]. It also allows comparing the functional outcome of TIO surgery to that of other treatment options such as Ganz’s osteotomy [16], for which gait analysis studies have been performed [17-19]. Several decrements (e.g. in walking speed and hip flexion pull-off power) after Ganz’s osteotomy have been found [17,18]. Since the surgical approach is slightly different between TIO (usually a modified Smith-Peterson approach) and Ganz’s osteotomy (usually either a classic Smith-Peterson or an abductor-sparing direct anterior approach), abnormalities in gait parameters after these respective operations may be related to the approach. Furthermore, the identification of specific gait and strength deficits might be useful for improving surgical techniques or postoperative rehabilitation protocols that may further enhance the functional outcome.

This study aimed to investigate the long-term outcome of TIO in terms of biomechanics of gait and muscle strength. Specifically, our objectives were to determine deviations in (1) spatiotemporal parameters, hip joint angles and joint moments during gait, and (2) hip and knee muscle strength in patients who had undergone TIO between two and ten years ago. We compared the results between the operated and non-operated limb and between patients and healthy controls.

## Methods

### Participants

Twelve women who had undergone TIO and eight healthy women in the same age range participated in this study. The indication for TIO was symptomatic hip dysplasia with a spherical femoral head, and without major damage to the cartilage [15]. All patients were operated in the Sint Maartenskliniek, Nijmegen, The Netherlands by the same experienced surgeon. The surgical procedure (modified Tönnis osteotomy) has been described in detail [20]. Patients between 18 and 70 years who had undergone a unilateral TIO between January 2003 and June 2010 were eligible for inclusion. This time window ensured that rehabilitation had been completed. Rehabilitation consisted of regular mobilization of the hip joint and strengthening exercises for the muscles of the hip region, and lasted for 3–12 months, depending on the patient’s individual speed of recovery. In total, 54 patients were screened, of whom 34 were eligible for inclusion. Of those, 18 patients were willing to participate, and 12 were included after screening for exclusion criteria. We excluded patients that had any disease or other condition that could affect their gait, including severe hip pain, as well as patients with a Body Mass Index (BMI) > 30 kg/m^2^ because of difficulties in marker placement for gait analysis. Selection was unspecific regarding gender, but all 12 included patients were women.

The study procedure was approved by the local ethical committee of the region Arnhem-Nijmegen, The Netherlands (study code 2012/065). A written informed consent was obtained from each subject.

### Clinical assessment

The participants were invited to the gait laboratory of the Radboud university medical center, Nijmegen, The Netherlands for a combined gait and muscle strength assessment session. We also obtained the Oxford [21,22] (range 0–48) and Harris Hip Score [23] (range 0–100) from the patients during a break in the session.

### Gait analysis

The participants walked barefoot on an 8 m long walkway at self-selected comfortable walking speed. An integrated data collection was performed including three-dimensional motion capture with synchronized force plate recordings. A six-camera digital optical motion capture system (Vicon MX, Oxford, UK) was used to record the position of 35 retro-reflective markers placed on the lower limb and torso (100 Hz). The standard Vicon Plug-in-Gait marker set was used, with additional markers placed on the anterior side of the thigh and lower leg at 1/3 and 2/3 segment length, and on the fifth metatarsal head of the foot. Two custom-made force plates (AMTI, Watertown, MA, USA), embedded level in the laboratory floor measured ground reaction forces (1000 Hz) during the stance phase of the gait cycle.

No specific instructions were given other than ‘walk naturally’ to prevent participants from targeting the force plates. Trials were repeated until six successful trials had been recorded, where ‘successful’ was defined as a trial in which each foot cleanly struck one of the two force plates. The gait analysis data of one patient and one control subject had to be discarded due to technical problems, leaving a group of 11 patients and 7 controls available for the gait analysis part of the study.

### Data analysis

Heel strike and toe off events were identified using thresholding of ground reaction force data (heel contact when F > 20 N, toe off when F < 20 N). Spatiotemporal parameters were subsequently calculated based on a combination of these events and the heel and toe marker position data.

A 21 degrees of freedom kinematic model (‘GaitLowerExtremityModel’, as available in the AnyBody Managed Model Repository 1.5.1) consisting of trunk, pelvis, thigh, shank, talus and foot segments was scaled to each subject based on the marker trajectories using the AnyBody Modeling System (version 5.3.1, AnyBody Technology A/S, Aalborg, Denmark). The marker trajectories were initially filtered with a 5 Hz 2^nd^ order Butterworth low-pass filter. Force plate data were low-pass filtered at 12 Hz with a 2^nd^ order Butterworth filter. The model was based on the kinematic part of the Twente Lower Extremity Model dataset [24]. After this procedure, lower limb joint angles were obtained by solving the inverse kinematics using the optimized parameters. At each joint, ideal torque generators were added. The segment masses were scaled using common scaling laws [25], available in AnyBody. The model marker positions, segment lengths and knee joint axes were then optimized using a parameter optimization algorithm [26]. Finally, the kinematics and ground reaction forces were used as input for an inverse dynamic analysis [27], in which joint moments were calculated in the local (ISB [28]) reference frames.

Our outcome variables of interest were spatiotemporal parameters, hip joint angles and moments in the sagittal and frontal planes. Each of these variables was averaged across trials to obtain subject ‘ensemble’ averages both for the operated and the non-operated limbs. The moments were normalized to the body weight (BW) and height (Ht) of the subject (%BW*Ht). In the sagittal plane, we determined the peak flexion and extension angles and moments. In the frontal plane, we used the adduction angle at 20% of the gait cycle in the analysis, since there was a peak in the angle at that particular phase of the gait cycle. Owing to the M-shaped curve of hip abduction moments during gait, we extracted two peak values, one in the first and one in the second half of the stance phase. For the healthy controls, we used the mean of the left and right limbs in the analyses.

### Muscle strength

**Figure 1:**
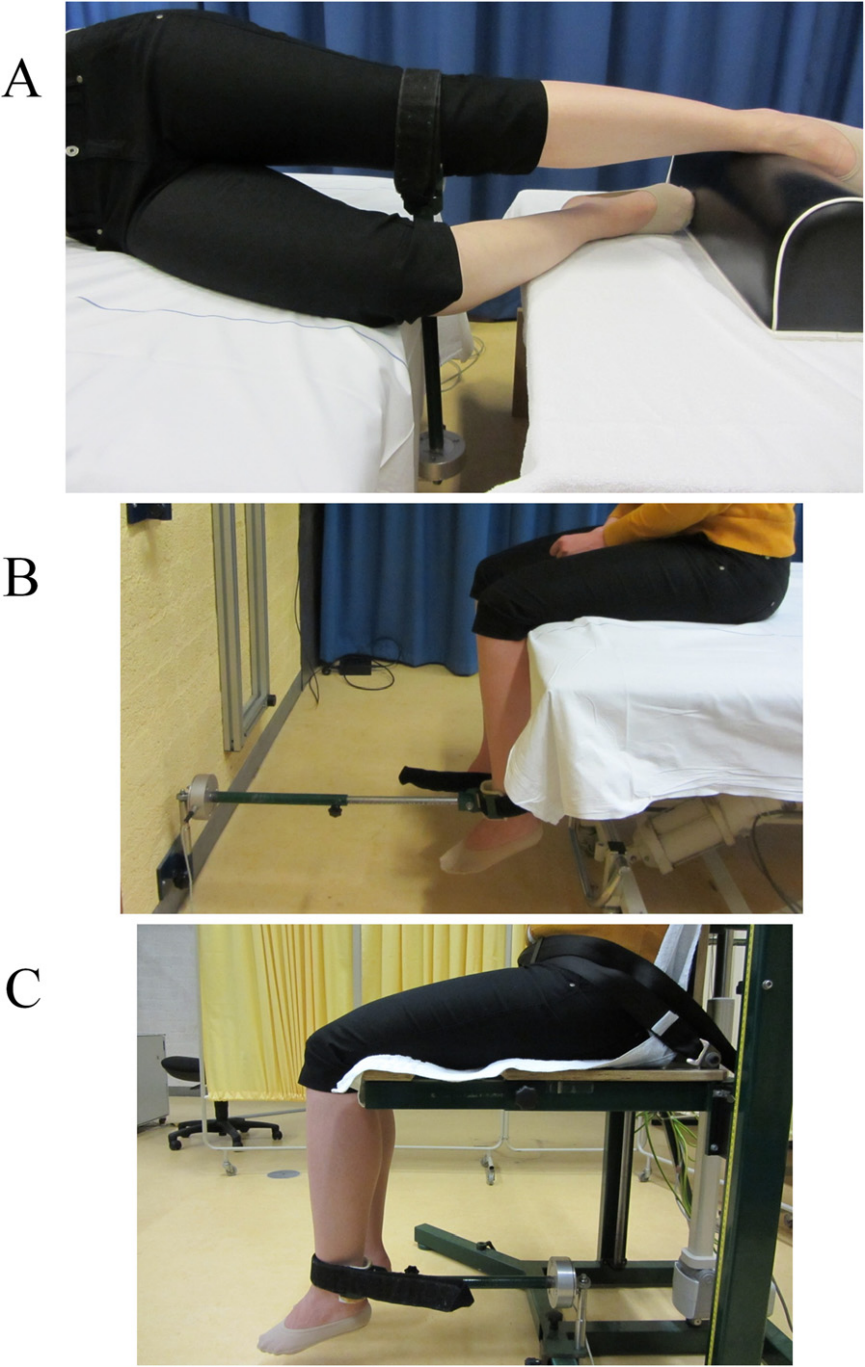
**Experimental setup for muscle strength measurements.** These were performed for **(A)** hip abduction, **(B)** knee flexion, and **(C)** knee extension. In all cases, the end piece (soft Velcro strap) of the force transducer was applied perpendicular to the limb, and the other end was rigidly attached to either a clinical testing bench, a wall, or a custom-built chair.

Isometric maximum voluntary contractions were recorded for hip abduction, and knee flexion and extension. Hip abduction strength was tested in side-lying position, with the tested hip at 0° flexion and 0° adduction, and the knee extended (Figure 1A). The non-tested hip was flexed at 45°, and the knee was flexed at 90° in order to prevent the contralateral limb from contributing to the maximum strength effort. The end piece (soft Velcro strap) of a force transducer was applied just proximal to the femoral epicondyles perpendicular to the limb, and the other end of the transducer was rigidly attached to the testing bench.

For maximum knee flexion strength testing, subjects sat on the edge of a testing bench positioned close to a wall (Figure 1B). The knee and hip were flexed at 90°, and the end piece of the force transducer was applied just proximal to the malleoli. The other end was rigidly fixed to the wall, level with the end piece. During the contractions, manual pressure was applied by an assistant proximal to the top of the tested knee to prevent it from rising. Knee extension was tested in a custom-built chair with vertical backrest, with the knee and hip flexed at 90°, and with the end piece of the force transducer applied just proximal to the malleoli (Figure 1C). The subject was strapped to the chair to prevent the waist from rising.

Participants performed three maximum isometric contractions for four seconds each, separated by 30 seconds of rest. All subjects received the same verbal encouragements during the contractions to achieve maximum effort. The highest recorded maximal force was multiplied by the moment arm to the joint centre of the tested joint to obtain peak torque values. Raw data was low-pass filtered at 6 Hz with a 2^nd^ order Butterworth filter and the peak torque was normalized by body weight.

### Statistical analysis

Student’s *t*-tests were used for comparing group characteristics, spatiotemporal parameters, kinematics and muscle strength tests between the patient and control groups. Paired samples *t*-tests were used for comparisons between operated and non-operated limbs. Hip joint moments during gait were tested with repeated-measures ANOVAs; one with phase of gait cycle (early and late stance) and limb (operated and non-operated) as within-subjects factors, and another with phase of gait cycle (early and late stance) as within-subjects factor and group (operated limb of patients and controls) as between-subjects factor. Post-hoc paired samples *t-*tests were used if the ANOVA indicated significant differences between the operated and the non-operated limb. The significance level was set at *P* ≤ 0.05. For the muscle strength data, significance was set at *P* ≤ 0.01 because of the number of *t*-tests performed (12). IBM SPSS Statistics 20.0.0.1 was used for all statistical analyses.

## Results

### Group characteristics

Descriptive characteristics of the patients who had undergone TIO and of the controls are shown in Table 1. No significant group differences were found for age, weight, height, or BMI. The participating patients were between 59 and 111 months post surgery. Their average Harris hip score was ‘good’ (84 points), and their average Oxford hip score was ‘excellent’ (42 points).

**Table 1:** **Characteristics of patients after triple innominate osteotomy and controls**

	**TIO patients n = 12**	**Controls n = 8**	P*
Age (years)	34 (12)	33 (10)	0.910
Weight (kg)	66.8 (5.2)	66.2 (11.6)	0.894
Height (m)	1.67 (0.07)	1.72 (0.07)	0.343
BMI (kg/m^2^)	23.9 (2.4)	22.3 (3.0)	0.232
Time since surgery (months)	80 (18)		
Operated limb	L: 3, R: 9		
Harris hip score	84 (15)		
Oxford hip score	42 (5)		

Data are presented as mean (SD).

*Student’s *t*-test for weight and BMI, Mann–Whitney U test for age and height.

### Spatiotemporal parameters

Spatiotemporal parameters are shown in Table 2. None of the parameters differed significantly between the groups. Within the patient group, the step length of the operated leg was 0.03 m smaller than that of the non-operated leg (*P* = 0.019), whereas the stance time was 1.2% longer (*P* = 0.039).

**Table 2:** **Spatiotemporal parameters of patients after triple innominate osteotomy and controls**

	**TIO patients**	**Controls**	P*
Walking speed (m/s)	1.32 (0.14)	1.34 (0.22)	0.752
Cadence (steps/min)	116.7 (5.5)	115.2 (13.1)	0.733
Step length operated limb (m)	0.66‡ (0.08)	0.69† (0.06)	0.386
Step length non-operated limb (m)	0.69‡ (0.05)	0.69† (0.06)	0.924
Stride length (m)	1.35 (0.12)	1.40 (0.11)	0.477
Stance duration operated limb (% of gait cycle)	61.2§ (2.1)	60.0† (1.5)	0.202
Stance duration non-operated limb (% of gait cycle)	60.0§ (2.0)	60.0† (1.5)	0.961

Data are presented as mean (SD).

*Student’s *t*-test.

†Mean of both legs.

‡Significantly different between operated and non-operated limb (paired *t*-test: *P* = 0.019).

§Significantly different between operated and non-operated limb (paired *t*-test: *P* = 0.039).

### Kinematic gait parameters

**Figure 2:**
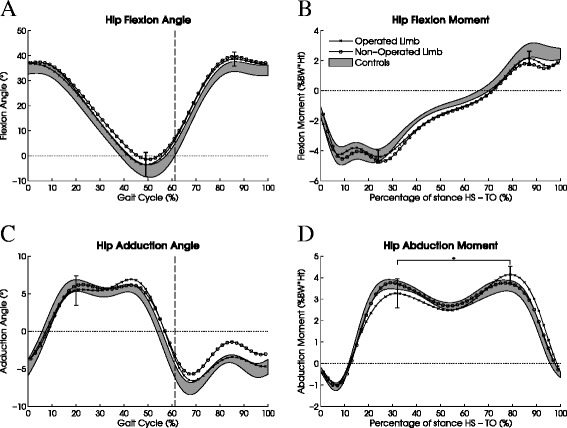
**Hip kinematics and kinetics of patients after triple innominate osteotomy and healthy controls.** Shown are **(A)** sagittal plane kinematics, **(B)** sagittal plane kinetics, **(C)** frontal plane kinematics, and **(D)** frontal plane kinetics. The kinematic data are normalized to full gait cycle (heel strike to ipsilateral heel strike); the kinetic data are normalized to the stance phase of the gait cycle (heel strike to ipsilateral toe off). Standard deviations of the mean of the operated limb are shown at the time points that were tested statistically (maximum extension angle, maximum flexion angle, hip adduction angle at 20% of the gait cycle, maximum extension moment, maximum flexion moment, and maximum hip abduction moment in early and late stance). The shaded areas represent equal boundaries of ±1SE for controls. The horizontal bracket and star in **(D)** indicate the significant interaction effect between the operated and the non-operated limb during the stance phase.

The kinematics of the operated and non-operated limb did not differ significantly from controls (Figure 2A and C). There were also no differences between the operated and non-operated limbs.

### Kinetic gait parameters

Hip abduction moments during stance are shown in Figure 2D. The operated limb had a different abduction moment pattern than the non-operated limb during the stance phase, as demonstrated by a significant *phase x limb* interaction effect (F_1,10_ = 5.78, *P* = 0.037); in early stance, the abduction moment in the operated limb was non-significantly lower than in the non-operated limb (*P* = 0.129), whereas in late stance it was non-significantly higher (*P* = 0.192). The main effect of *limb* was not significant (F_1,10_ = 0.12, *P* = 0.742). The operated limb was not significantly different from controls (*group:* F_1,16_ = 0.00, *P* = 0.949; *phase x group:* F_1,16_ = 2.63, *P* = 0.125).

Sagittal plane hip moments during stance are shown in Figure 2B. The hip flexion and extension moments generated by the operated limb of the patients were not different from the unaffected limb (*limb:* F_1,10_ = 0.03, *P* = 0.861, *phase x limb:* F_1,10_ = 1.51, *P* = 0.247). The operated limb did not differ from controls (*group:* F_1,16_ = 2.62, *P* = 0.125, *phase x group:* F_1,16_ = 0.28, *P* = 0.601).

### Muscle strength

**Figure 3:**
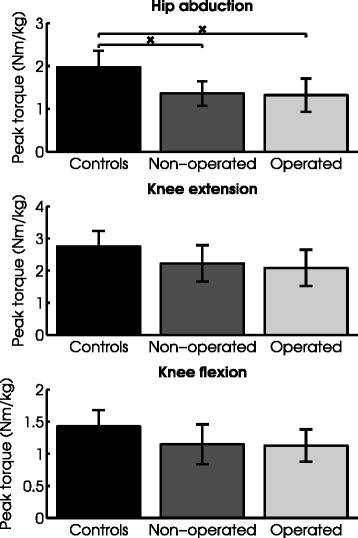
**Maximum generated torque of patients who had undergone triple innominate osteotomy and controls.** Peak torque values are given in Newton-meters, normalized to body weight. The error bars represent equal boundaries of ±1SD. ^x^ Indicates a statistically significant difference (*P* ≤ 0.01).

Abduction strength was significantly lower than healthy controls in both the operated and the non-operated limb (Figure 3), whereas differences in knee flexion and extension strength between patients and controls did not reach significance. The operated and non-operated limbs did not differ in any of the strength measurements.

## Discussion

In this study, we found that patients who had undergone TIO (on average 80 months ago) did not have major abnormalities in their gait pattern compared to controls, but did exhibit subtle asymmetries between the operated and non-operated limbs. The operated limb had a smaller step length and longer stance time compared to the non-operated limb. In addition, the abduction moment pattern during the stance phase was significantly different between the operated and non-operated limbs. In terms of muscle strength, a bilateral abduction strength deficit was found. Muscle strength did not differ between the operated and the non-operated limb for any of the tested joints.

Interestingly, we found subtle asymmetries in hip abduction moments between the operated and non-operated limbs. This may represent a more cautious walking pattern, by enabling a more gradual transfer of the body’s centre of mass over the operated limb, thereby reducing the peak hip abduction moment in early stance. This does not seem to be related to a lack of hip abductor strength, as there was no difference in strength between the operated and non-operated leg. The reduced step length and prolonged stance duration that we found in the operated limb, albeit rather small, may also point to an integral strategy in the frontal plane reflecting a more cautious walking pattern.

Compared to healthy controls, we found significantly reduced hip abduction strength in patients who had undergone TIO. Hip abductor weakness is a common finding in patients with hip dysplasia, frequently resulting in Trendelenburg’s sign during gait [2,29]. Hence, the persisting weakness that we found may be reflective of severe muscle atrophy that had developed over many years prior to surgery and that did not fully recover despite postoperative rehabilitation. Alternatively, our patient group may have been less physically active than the controls, resulting in poorer muscle strength. We indeed observed a general trend of muscle strength reduction in all the tests performed, but the pronounced weakness in hip abduction argues against an overall reduction in physical activity being the only responsible factor.

Our finding that hip abduction strength was substantially lower in patients compared to controls, whilst no main effect of group could be demonstrated for hip abduction moments during gait implies that the patients walked at a higher percentage of their maximum capacity. This may eventually result in gait pattern changes when patients become fatigued, as it has been shown that human gait is relatively sensitive to weakness in the hip abductor muscle group [30]. A gait pattern typical of abductor weakness (Trendelenburg’s sign) may then emerge, involving pelvic drop at the contralateral side. This increases hip joint stress in the operated hip due to a decrease in joint contact area [31], which may speed up the development of osteoarthritis in the operated hip.

The clinical results of TIO at comparable follow-up times are generally ‘good’, ‘excellent’, or ‘improved compared with before the operation’ [1,4,32], which is congruent with the results of our study. Hence, the functional status of our study population appears to be representative of the TIO group at large. To our knowledge, up to this point no studies have been published that employed gait analysis or maximum strength measurements to describe the functional outcome after TIO surgery. In contrast, results from gait analyses have been reported for patients after Ganz’s periacetabular osteotomy [16], which conceptually is a rather similar procedure to the modified Tönnis osteotomy used here [20]. Our findings that patients after TIO did not demonstrate major gait deviations compares well to previous results from patients after Ganz’s osteotomy. After that surgery, no statistically significant differences from controls could be demonstrated in sagittal hip joint kinematics and kinetics [19,33] or frontal plane kinetics [18]. Similarly, Karam et al. investigated spatiotemporal gait parameters before and one year after Ganz’s osteotomy [17] and found post-operative walking velocities, stride lengths and cadences very similar to our results. Thus, the relatively small differences in the surgical approach between TIO and Ganz’s osteotomy (even if it was abductor-sparing, as in the case of Sucato et al. [18]) do not appear to play a major role in long-term functional outcome.

Our study has certain limitations. First, we invited 34 patients to participate in the study, but only 18 responded, out of which 12 patients could be included. The non-responders and excluded patients might have had more pain or functional limitations than those included. Moreover, since there were only women in our study population, the results might not apply to men who underwent TIO. Second, although the study groups were similar with regard to age, gender, height, weight and BMI, we did not take confounding factors into account such as general activity level or participation in sports. As such, it is possible that the patients were less active than the controls and that, therefore, they had less muscle strength around the hip and knee joints. Third, we did not perform gait and strength measurements before the surgery. Such analyses could reveal whether the deviations in gait observed post-operatively in this study stem from pre-operatively adopted gait patterns. Fourth, we did not adjust the geometry of the operated hip joint in the AnyBody model. The joint’s centre of rotation in our patients might have been slightly altered by the TIO, thereby influencing the joint moment calculations.

## Conclusions

This study showed that patients who underwent TIO generally recovered well from their operation with regard to gait, but did exhibit subtle asymmetries between the operated and non-operated limbs several years after surgery. Muscle strength deficits were found bilaterally in hip abduction. Although the differences observed in gait were subtle, they may become clinically important in young or active patients who engage in high-level activities over many years. Thus, in further research in this patient group it is recommended to focus on gait adaptations after prolonged walking.
